# Successful Endoscope-Assisted Transcervical Insemination (TCI) in Dogs Using Sperm Recovered from Epididymides Stored at 5 °C for 24 h After Castration Prior to Semen Collection and Cryopreservation

**DOI:** 10.3390/vetsci13040326

**Published:** 2026-03-27

**Authors:** Mónika Bacsa, Eszter Szilágyi, Kristián Erdei, Linda Müller, Eszter Nagy, Balázs Attila Dobos, Tamás Radovits, Sándor Cseh

**Affiliations:** 1 Department of Obstetrics and Food Animal Medicine Clinic, University of Veterinary Medicine, 1078 Budapest, Hungary; szilagyi.eszter@univet.hu (E.S.); erdei.krisztian@univet.hu (K.E.); muller.linda@univet.hu (L.M.); nagy.eszter@univet.hu (E.N.); cseh.sandor@univet.hu (S.C.); 2 Heart and Vascular Center, Faculty of Medicine, Semmelweis University, 1122 Budapest, Hungary; dobosattilabalazs@gmail.com (B.A.D.); radovitstamas@yahoo.com (T.R.)

**Keywords:** cryopreservation, frozen-thawed epididymal semen, transcervical intrauterin insemination

## Abstract

The recovery and cryopreservation of epididymal spermatozoa enable genetic preservation in male dogs that die unexpectedly or require castration; however, sperm collection is typically performed immediately after surgery, and artificial insemination is often surgical. This study aimed to evaluate whether epididymides stored at 4–5 °C for 24 h prior to sperm recovery retain fertilizing capacity after cryopreservation and whether pregnancy can be achieved using endoscopically guided transcervical intrauterine insemination. Testes and epididymides from an eight-month-old German Shepherd dog were stored in physiological saline at 4–5 °C for 24 h following castration. Spermatozoa were recovered from the cauda epididymis using single-incision aspiration, evaluated, frozen according to the Uppsala method, and stored in liquid nitrogen for two months. After thawing, 62 × 10^6^ progressive motile spermatozoa were inseminated once into a bitch in heat using transcervical endoscopic guidance. Pregnancy was confirmed by ultrasonography, and nine healthy puppies were delivered at term. These findings demonstrate that 24 h refrigerated storage of the epididymis does not impair post-thaw fertilizing ability and that non-surgical transcervical intrauterine insemination represents an effective alternative to surgical techniques for the use of frozen–thawed epididymal semen in dogs.

## Simple Summary

Epididymal sperm collection followed by cryopreservation allows the production of offspring even after the male is no longer reproductively available. In most published studies, sperm is collected immediately after castration, and insemination is typically performed surgically under general anesthesia. In this study, we report the birth of nine healthy puppies following insemination with frozen–thawed epididymal sperm recovered from organs stored at 4–5 °C for 24 h prior to sperm collection. The spermatozoa were cryopreserved for two months and subsequently deposited into the uterus using the endoscopically guided transcervical technique that does not require surgery or general anesthesia. Despite the presence of a moderate number of progressively motile sperm cells after thawing, pregnancy was achieved after a single well-timed insemination. These findings indicate that short-term refrigerated storage of the epididymis is a practical option when immediate processing is not possible and that non-surgical intrauterine insemination represents an effective and welfare-friendly approach in dogs.

## 1. Introduction

The recovery and cryopreservation of epididymal sperm are important tools for preserving the genetic material of valuable breeding animals and endangered species, particularly when sperm cannot be collected using conventional methods or after death. Epididymal sperm can be retrieved using various techniques, including percutaneous or microsurgical aspiration in human reproductive medicine, and float-up or retrograde flushing methods in veterinary practice. These approaches are commonly applied after castration or death to enable the preservation of genetic resources for future use. The recovered sperm can subsequently be cryopreserved and utilized in assisted reproductive techniques, such as artificial insemination (AI) [1,2].

Canine epididymal sperm has great potential for preserving the genetic material of males who die unexpectedly or suffer from illnesses that negatively impact male fertility. Such illnesses can range from severe prostatic problems to paralysis and back pain. In any situation where the male must be castrated, sperm collection from the epididymis and storage can be a good option for preserving and maintaining these dogs’ reproductive potential. For valuable males that must be euthanized for welfare reasons, harvesting and storing sperm can provide a final chance to produce offspring in the future.

In 1994, Marks et al. published their findings on the successful use of frozen-thawed epididymal sperm for surgical intrauterine insemination to achieve pregnancy, resulting in the birth of one viable female pup. The male was euthanized, and his testicles were removed one hour after death [3]. Kim et al. achieved a 23.8% pregnancy rate using frozen–thawed epididymal semen. AI was carried out by inserting a transcervical catheter into the uterine body; however, the whelping rate and litter size were not reported [4]. Hori et al. achieved similar pregnancy rates using the intrauterine (16/1, 6.3%) and intratubal laparotomy (6/1, 16.7%) methods. They harvested the epididymal spermatozoa immediately after surgical excision [5]. Their study suggests that missing prostatic fluid is the cause of low pregnancy rates. Hori et al. used prostatic fluid to sensitize epididymal semen after collection prior freezing and achieved an 80% pregnancy rate with surgical intrauterine insemination compared to a 20% pregnancy rate with unsensitized semen [6]. They also demonstrated that semen is suitable for cryopreservation and spermatozoa can be recovered from epididymides that were stored at 4–5 °C for 48 h prior to semen collection. Frozen epididymal sperm was treated with prostatic fluid after collection. The surgical insemination with laparatomy was carried out under general anesthesia. Surgical inseminations resulted in pregnancy in four out of five bitches (80%) [7]. In a subsequent study, however, only a 12.5% conception rate was obtained with the same collection method (collection was performed right after castration) and AI technique, but in this clinical trial, the bitches had only one ovary [8]. Axnér and Hermansson [9] recently published that they had achieved pregnancy and living pups with endoscopically guided transcervical intrauterine insemination using frozen epididymal semen. The semen was collected from a 7-year-old hunting dog that had to be euthanized because of serious injuries. Castration was performed on the dog prior to euthanasia in order to harvest the epididymal spermatozoa by mincing the cauda epididymis. There were eight pups born 59 days following TCI [9]. Research by Kim et al. and Axnér and Hermansson [4,9] shows that there are other methods that can be used for conception using frozen epididymal sperm outside surgical insemination.

Based on the work of Klinc et al. and Wydooghe et al., it is proven that fresh and chilled epididymal sperm, as well as frozen-thawed epididymal sperm, possess fertilizing capacity [10,11].

Intravaginal, surgical intauterin, and TCI methods can be used depending on the type of semen. If using frozen semen, it is recommended to deposit it into the female dog’s uterus because the frozen semen has a shorter lifespan than fresh or chilled semen [12]. But surgical insemination is an invasive technique and requires general anesthesia, in contrast to endoscopically guided transcervical intrauterine insemination. In several countries, surgical insemination is already prohibited because of animal welfare reasons [13].

To our knowledge, this is the first paper to report that a bitch was successfully inseminated with sperm collected from epididymides that had been stored at 5 °C for 24 h prior to sperm extraction, resulting in the birth of healthy puppies. The collected epididymal spermatozoa were cryopreserved and stored for two months in liquid nitrogen until TCI.

Our results indicate that storing epididymis at 4–5 °C for 24 h prior to sperm collection has no negative effect on the deep freezability of the extracted spermatozoa, or on its fertilization capacity after thawing. Storage at 4–5 °C may be a solution for collecting epididymis from unexpectedly deceased animals, transporting epididymis from castrated animals over longer distances, and organizing work related to sperm collection and deep freezing.

## 2. Detailed Case Description

In September 2025, an eight-month-old, 25 kg male German Shepherd dog was castrated. The castration took place in the Obstetrics Clinic of the University of Veterinary Medicine, Budapest, as part of the neutering program. The semen donor male dog was kept in a shelter house until operation.

After castration, the testicles and related epididymis, covered by the tunica vaginalis, were placed in physiological saline solution (Salsol Inf.) and stored in a refrigerator at 4–5 °C for 24 h [14].

The organs were delicately withdrawn from the saline solution and dried with a clean paper towel before sperm collection. The testes and the epididymis were made visible by cutting the tunica vaginalis. The plexus pampiniformis was cut to prevent bleeding. If it was possible, a testicularis was also separated from the ductus. The epididymis was then dissected from the testis together with a 3–4 cm segment of the ductus deferens. The cauda epididymis was subsequently cleared of surrounding capillaries, and residual blood was gently removed using sterile paper towels.

**Figure 1:**
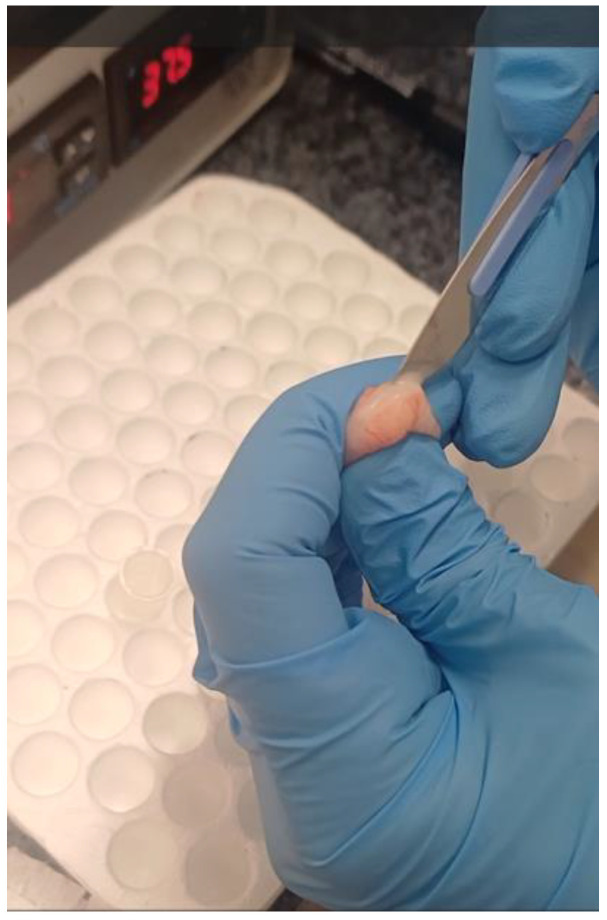
Epididymal sperm collection with the single-incision sperm aspiration (SESA) technique. A single incision was made in the cauda epididymis, and the emerging sperm-rich fluid was aspirated and transferred into prewarmed PBS.

Sperm was collected using the SESA (single-incision sperm aspiration) technique in order to minimize blood content and tissue fragment contamination [2,5,15]. Cauda epididymis was fixed and held tightly, while a single incision was made with a sterile scalpel blade, and the resulting white droplets containing spermatozoa were aspirated with an automatic pipette and placed into 2 mL eppendorf containing prewarmed (37 °C) 1 mL PBS. The proximal part of the ductus was also cut, and the content was aspirated. Sperm was collected from both epididymides (Figure 1).

The semen was evaluated a few minutes later. Motility and concentration were evaluated with CASA (Microptic, Barcelona, Spain), and the phase-contrast microscope’s CASA settings were as follows: Sperm cells were classified as medium progressive at velocities of 65–100 µm/s and rapid progressive at velocities >100 µm/s. Progressive motility (sum of medium and rapid progressive cells) was defined by a straightness (STR) value >75%. Analyses were performed at a frame rate of 50 frames per second (fps), with a minimum of 500 spermatozoa evaluated across at least five fields. A 10 µL well-mixed semen sample was placed on a heated slide and covered with a cover slip. The motility parameters were as follows: 23.11% progressive motility, 86.22% total motility. Smears were prepared to examine morphology. Morphology was assessed with Spermac stain. In total, 50% of the cells exhibited normal morphology, 40% had distal cytoplasmic droplet, and 10% showed acrosomal defect. A total of 372 × 10^6^ spermatozoa were counted in the sample.

The semen was frozen using the Uppsala method [16]. Briefly, sperm cryopreservation was performed using the two-step Uppsala protocol. Initially, semen samples were diluted with extender 1 and equilibrated for 75 min at 4–5 °C. Subsequently, extender 2 was added, and after a short equilibration period of a few minutes, the samples were loaded into 0.5 mL plastic straws. The filled straws were placed horizontally on a rack positioned approximately 4 cm above the level of liquid nitrogen and exposed to nitrogen vapor for 10 min. The samples were loaded into 0.5 mL straws. Three and a half straws were filled (the concentration of the straws was 200 M/mL). A half-filled straw was reserved for testing. Thawing was performed in a water bath at 37 °C for 60 s. The sample was placed on a heated stage set to 37 °C and evaluated after a few minutes. Progressive motility was 19.59%, and total motility was 93.06%, indicating that there were 62 × 10^6^ progressive motile sperm cells in the insemination dose.

**Figure 2:**
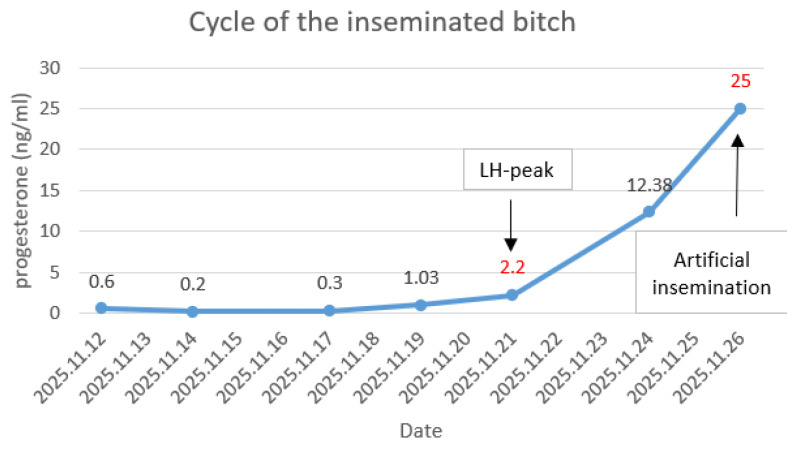
Cycle monitoring and timing of insemination. Progesterone levels were measured repeatedly using a chemiluminescence immunoassay (CLIA). LH peak was defined at 2 ng/mL and ovulation at 5 ng/mL progesterone concentration. Transcervical intrauterine insemination was performed once, on day 5 after the LH peak.

In 2025 November, a two-year old foxhound female dog began showing signs of heat. The inseminated female dog was kept in the animal care facility of the Heart and Vascular Clinic under 24 h video surveillance. She had unlimited access to water and was fed dog food twice a day. For cycle monitoring, blood samples were taken on several occasions to measure the progesterone level. The progesterone level was measured with the CLIA method (Chemiluminescence Immunoassay). A progesterone level of 2 ng/mL was evaluated as LH peak, and 5 ng/mL was evaluated as ovulation (Figure 2).

On the 5th day after the LH peak, artificial insemination was performed once with the transcervical intrauterine endoscopic method. All frozen straws were thawed and used for insemination, and no prostatic fluid was added to the insemination dose. On the day of the TCI, the progesterone level was 25 ng/mL. A MOFA Medium Fiberoptic TCI device set (consisting of an endoscope and associated accessories) (MOFA, USA) was utilized for insemination. No sedation was necessary during the AI procedure, and no postoperative care was required afterwards. An ultrasound pregnancy test was performed on days 21 and 29 after the artificial insemination to confirm the pregnancy, and several live fetuses were found. On the 59th day after LH peak, radiography revealed nine well-mineralized fetuses. On the 65th day after LH peak, nine healthy puppies were born (three females, six males).

The animals were not selected based on predefined criteria but represented clinical cases.

The protocol of the animal experiment was approved by the Food Chain Safety and Animal Health Directorate of Pest County’s Government Office (PE/EA/00825-3/2023).

## 3. Discussion

In most studies involving insemination with epididymal dog sperm, surgical AI was used, which requires anesthesia, postoperative care, and medication [3,5,7,8]. Only a few authors have written about AI using non-invasive insemination methods such as intravaginal insemination or transcervical catheterization [4,9,10,11]. If we focus only on insemination using frozen-thawed epididymal semen, there are only a few publications about non-invasive methods, such as TCI [4,9]. Both techniques are used in veterinary facilities for ejaculated semen. However, the surgical method is banned in some countries, such as the United Kingdom, Ireland, Australia, and Scandinavia, due to animal welfare concerns [13,17,18]. Retrospective clinical studies have shown that fertility rates achieved via TCI are the same as or higher than those achieved via surgical AI in dogs, particularly when frozen–thawed ejaculated semen is used [19,20]. Based on these findings, the TCI technique may also be recommended for artificial insemination using epididymal semen.

The total sperm count in studies involving AI using frozen–thawed epididymal semen varies from 51.5 × 10^6^ motile sperm cells to 217 × 10^6^ progressive motile cells. Hori et al. [5,6,7,8] used typically 200 × 10^6^ cells for AI, but the number of motile or progressive motile cells was not specified. The study by Kim et al. provided no information on concentration or total cell count [4]. Mason et al. found a significantly higher pregnancy rate when >150 × 10^6^ progressive motile cells (76%) or 100–150 × 10^6^ progressive motile cells (68%) were inseminated compared to <100 × 10^6^ cells (52%). These results indicate that good pregnancy rates can be obtained with one, well-timed insemination via transcervical intrauterine endoscope using >100 × 10^6^ progressive motile spermatozoa [19]. In the present case, the number of progressive motile spermatozoa after thawing was relatively low (62 × 10^6^); however, pregnancy was successfully achieved, resulting in the birth of nine healthy puppies. This favorable outcome may be explained by the precise timing of insemination relative to the LH peak, as the procedure was performed on day 5 after the LH peak, in accordance with the recommendations of Cochran et al. [21]. These findings highlight the importance of accurate reproductive timing, which may compensate for a lower sperm dose. The pregnancy rates of AI in frozen–thawed epididymal studies vary from 0 to 80% [5,6,7,8].

In our study, after castration, the extracted organs were placed in physiological saline solution (Salsol inf.) for 24 h and kept at 4–5 °C prior to the spermatozoa collection and freezing. In most studies, the spermatozoa collection, evaluation, and handling occurred on the same day as the castration, a couple of minutes or hours after the operation [2,3,9,22,23,24,25]. In one study, sperm cells were harvested on the same day as the castration, but the cells were taken into an extender and stored at 5 °C for 2 and 4 days before freezing [26]. There are some studies in which the epididymal spermatozoa were collected and evaluated after the organs underwent chilled storage for 12, 24 and 48 h, but with these samples, AI was not performed. However, these experiments clearly demonstrate that storing the organs in physiological saline in a refrigerator for 24 h is an effective method of maintaining semen quality, based on motility, viability, acrosome integrity, and normal morphology results. It is better to store the organs in a refrigerator (chilled condition) compared to room temperature, especially for a longer time before handling [15,27,28]. Hori at al. in 2005 published results in connection with intrauterine insemination with frozen–thawed epididymal sperm recovered from epididymides that were stored in a refrigerator for 48 h, and they obtained an 80% pregnancy rate (four out of five bitches became pregnant). A very important finding of this paper is that although the motility results of epididymal sperm cells stored at 4 °C were significantly lower compared to those that were not exposed to low temperatures (spermatozoa were immediately collected after excision and also in the case of ejaculated samples), but no significant differences were observed in motility results among the groups after freezing–thawing. This means that only cold-resistant cells retain their motility after freezing and thawing, regardless of when the organs were processed—on the same day as the castration or 24–48 h after being stored in a refrigerator. In this publication, the authors proved that it is possible to produce offspring using frozen–thawed epididymal sperm collected from organs stored at 4–5 °C for 48 h. However, in contrast to our study, they used surgical intrauterine AI [7].

The sperm collection technique is another process that has been investigated. Many authors have used the mincing method, which is a very effective method because a high total cell count and good quality semen can be obtained, but the disadvantage of the method is the contamination of the samples with blood and tissue fragments [5,15,22]. Haemospermia negatively affects the quality of the frozen–thawed semen [29,30]. A metal mesh is recommended to be used to filter the transmigrated sperm to remove tissue fragments, but this may cause cell loss. Single-incision epididymal sperm aspiration (SESA) is a reliable method for harvesting good quality semen without blood and tissue contamination, but a lower number of cells are collected with this technique [29]. This approach was considered advantageous in the present case, as it allowed the recovery of sperm with minimal contamination while maintaining adequate quality for cryopreservation and successful insemination.

## 4. Conclusions

In our study, pregnancy was achieved using frozen–thawed epididymal spermatozoa recovered from epididymides stored at 4–5 °C for 24 h post-castration. Spermatozoa were collected using the SESA method and inseminated via endoscope-guided transcervical intrauterine insemination (TCI). These findings indicate that epididymal sperm retains fertilizing capacity following short-term refrigerated storage prior to collection and cryopreservation. Refrigerated storage of excised organs for up to 24 h may therefore represent a practical approach for workflow management or when prolonged transport to the laboratory is required. Based on our results, TCI appears to be a suitable method for artificial insemination with epididymal semen, potentially reducing the need for more invasive surgical techniques.

This study has some limitations. As a single case report, the findings cannot be generalized, and further studies involving a larger number of animals are required to confirm the reproducibility and broader applicability of the results. Nevertheless, the present case provides clinically relevant evidence supporting the feasibility of the described approach.

## Data Availability

The original contributions presented in this study are included in the article. Further inquiries can be directed to the corresponding author.

## Abbreviations

The following abbreviations are used in this manuscript:

AI	artificial insemination
TCI	transcervical insemination
SESA USA	single-incision epididymal sperm aspiration United States of America
